# Macrophage targeting contributes to the inhibitory effects of embelin on colitis-associated cancer

**DOI:** 10.18632/oncotarget.6969

**Published:** 2016-01-21

**Authors:** Ting Wu, Yun Dai, Weihong Wang, Guigen Teng, Hongmei Jiao, Xiaowei Shuai, Rongxin Zhang, Peng Zhao, Liang Qiao

**Affiliations:** ^1^ Department of Gastroenterology, Peking University First Hospital, Beijing 100034, China; ^2^ Department of Gerontology, Peking University First Hospital, Beijing 100034, China; ^3^ Research Center of Basic Medical Sciences and Department of Immunology, Key Laboratory of Immune Microenvironment and Diseases of Educational Ministry of China, Tianjin Medical University, Tianjin 300070, China; ^4^ Department of Colorectal Cancer, Tianjin Medical University Cancer Institute and Hospital, National Clinical Research Center for Cancer, Key Laboratory of Cancer Prevention and Therapy, Tianjin 300060, China; ^5^ Storr Liver Centre, The Westmead Institute for Medical Research, The University of Sydney at Westmead Hospital, Westmead, NSW 2145, Australia

**Keywords:** embelin, colitis-associated cancer, macrophage, target therapy

## Abstract

Macrophages are a major component of inflammatory and tumor microenvironment. We previously reported that embelin suppresses colitis-associated tumorigenesis. Here, the role of macrophage targeting in the anti-inflammatory and anti-tumor properties of embelin was investigated. By using colitis-associated cancer (CAC) model, we demonstrated that embelin significantly depleted colon macrophages by blocking their recruitment. Moreover, embelin attenuated M2-like polarization of macrophages within the tumor microenvironment and eliminated their tumor-promoting functions during the development of CAC. Embelin potently inhibited NF-κB signaling in macrophages and decreased the production of key pro-inflammatory cytokines and tumorigenic factors involved in CAC, such as TNFα, IL-6 and COX-2. In addition, embelin directly reduced the polarization of M2 macrophages *in vitro* even in the presence of Th2 cytokines. These results suggested that targeting macrophages is, at least in part, responsible for the anti-tumor activity of embelin in CAC. Our observations strengthen the rationale for future validation of embelin in the prevention and treatment of CAC

## INTRODUCTION

Chronic inflammation plays a promoting role in the initiation and progression of many cancers, and as such inflammation is now regarded as one of the important hallmarks for cancer [1]. It is now believed that the inflammatory microenvironment is an essential component of tumor formation and growth [2]. The role of chronic inflammation in cancer development is very well-reflected in the clinical studies showing an elevated risk of developing colorectal cancer (CRC) in patients with inflammatory bowel disease (IBD) such as ulcerative colitis and Crohn's disease [3, 4]. The risk of CRC increases with the duration and anatomic extent of colitis, whereas it decreases when patients are treated with anti-inflammatory agents such as mesalamine [5]. Unlike the sporadic CRC, colitis-associated caner (CAC) follows a different histological sequence, starting in the inflamed mucosa as a hyperplastic lesion and evolving through dysplasia into adenocarcinoma. This event is widely known as the “inflammation-dysplasia-carcinoma” sequence [6].

Macrophages are a major component of the chronic inflammatory and tumor milieu. They create a microenvironment that is mutagenic and can promote tumor initiation [1, 7, 8]. In a mouse model of CAC, macrophages were found to drive tumor progression through the production of pro-inflammatory cytokines, such as tumor necrosis factor α (TNFα) and interleukin 6 (IL-6), which are well-known for their pro-proliferative effects on cancer cells *via* nuclear factor-κB (NF-κB) and signal transducer and activator of transcription 3 (STAT3) signaling [9–11]. As tumors progress to malignancy, tumor-associated macrophages (TAMs) support angiogenesis, enhance tumor cell migration and invasion, and suppress anti-tumor immune responses [8]. Macrophages can be categorized into “classically activated” M1 and “alternatively activated” M2 subtypes based on their polarization status. M1 macrophages can be activated by Th1 cytokine interferon γ (IFNγ) and microbial products, and they express high levels of pro-inflammatory cytokines (e.g. TNFα, IL-1, IL-6, IL-12 and IL-23), and inducible nitric oxide synthase (iNOS), and are capable of killing pathogens and tumor cells. In contrast, M2 macrophages that differentiate in response to Th2 cytokines such as IL-4, IL-10 and IL-13, show increased expression of IL-10, scavenger receptor and arginase [7, 8]. In the context of TAMs, M1 macrophages are considered to exert tumoricidal effects whereas M2 macrophages promote tumorigenesis. Both M1 and M2 TAMs are plastic and reversible, and the tumor microenvironment plays a major role in the regulation of functional polarization of TAMs. Previous studies have shown that tumor progression is associated with a phenotype switch from M1 to M2 [12]. Conversely, the tumor-promoting M2 TAMs can be reversed to anti-tumor M1 phenotype by “re-educating” [13, 14]. As such, targeting TAMs may possess promise as a new option in anti-cancer intervention. Strategies of TAM-based targeted therapy include deletion, re-education, and modulation [15, 16]. Indeed, macrophage depletion has been shown to successfully limit tumor growth and metastasis, and therefore leading to a better response to conventional therapy [17].

Embelin (2,5-dihydroxy-3-undecyl-1,4-benzoquinone) is a potent, nonpeptidic, cell-permeable small molecule inhibitor of X-linked inhibitor of apoptosis protein (XIAP) [18]. We previously reported that embelin effectively suppresses colon carcinogenesis in mouse model, and inhibits the growth of colon cancer cells by reducing cell proliferation and inducing apoptosis. The anti-tumor effects of embelin could be partly attributed to its inhibition of NF-κB and STAT3 pathways [19, 20]. Moreover, embelin decreases the expression of cytokines such as IL-6, IL-1β and IL-17a, and reduces the infiltration of CD4^+^ T cells in the tumor stroma [20]. These data suggested that embelin may not only target the tumor cells but also affect the tumor microenvironment.

Given the key role of macrophages in chronic colitis and the associated tumorigenesis, in the present study, we aimed to elucidate the role of macrophage targeting in the anti-tumor activity of embelin by using a well-established CAC model. We particularly focused on the effects of embelin on modulating the phenotype and function of macrophages both *in vitro* and *in vivo*.

## RESULTS

### Embelin altered macrophage population during CAC development

**Figure 1:**
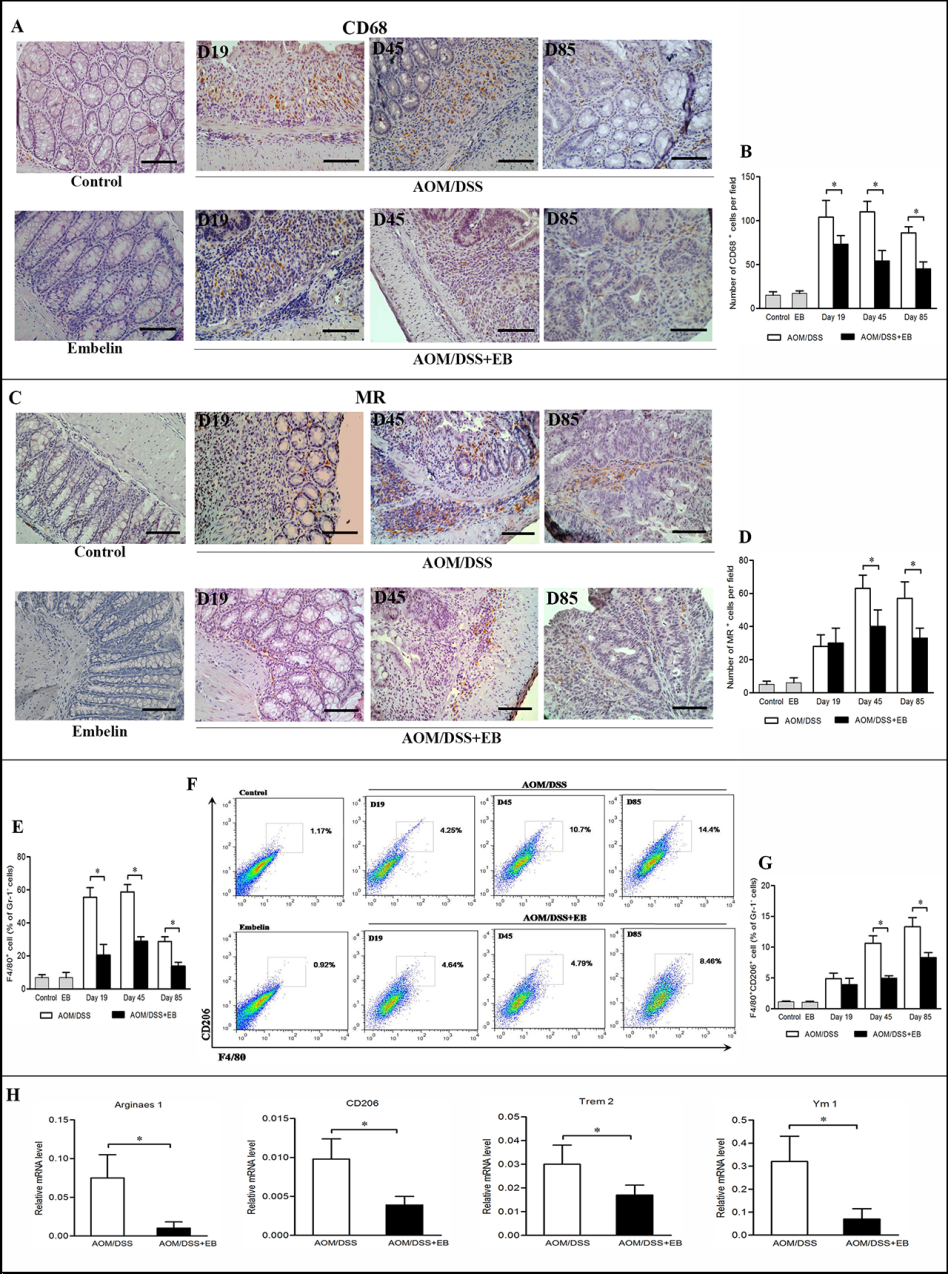
Embelin (EB) altered macrophage population during CAC development Immunohistochemical analysis of the pan-macrophage marker CD68 (**A**) and M2 macrophage marker MR (**C**) at days 19, 45 and 85 after AOM/DSS treatment. Scale bar: 50 μm. The number of CD68^+^ (**B**) and MR^+^ macrophages (**D**) per field was counted (*n* > 10 fields/group). For flow cytometric analysis, immunocytes were extracted from colonic tissues and stained with anti-F4/80, anti-CD206, and anti-Gr1 antibodies. (**E**) The percentage of F4/80^+^ macrophages among total Gr-1^−^ cells. (**F**) The blot shown was gated on Gr-1^−^ cells, F4/80 and CD206 flow plot was used to identify M2-like TAMs. F4/80^+^CD206+ macrophages among total Gr-1^−^ cells were quantified (**G**. **H**). Colon tissues were collected at day 85 after AOM/DSS administration and mRNA levels of M2 genes Arginase1, CD206, Trem2 and Ym1 were determined by qPCR. Data are expressed as mean ± SD (*n* = 6–8/group; *p < 0.05).

In the CAC model, embelin not only significantly resolved the inflammatory response of the colonic epithelium but also limited the colitis-associated tumor initiation and progression (Supplementary Data Figure S1 and our previous manuscript [20]). As macrophages play a pivotal role in inflammation and associated tumorigenesis, we explored the effects of embelin on macrophages within the colon tissue at different stages of CAC. To distinct the subtype of macrophages, CD68 was used as the common marker for macrophages and a well-established prototype marker mannose receptor (MR) was used for M2 macrophages by immunohistochemical analysis. Infiltration of macrophages was clearly shown in the colon tissues of CAC mice at the stages of acute colitis-repair (day 19), chronic colitis and dysplasia (day 45) and carcinoma (day 85) (Figure 1A). Increased infiltration of macrophages was observed in the entire colon of CAC mice including the relatively non-inflamed and non-dysplasia portions, as evidenced by an increased CD68 and MR staining. There was a marked reduction of the colonic infiltration by CD68^+^ macrophages in CAC mice treated with embelin at all time points (Figure 1A and 1B). Notably, infiltration by the MR^+^ macrophages was significantly diminished in response to embelin during the phase of dysplasia (day 45) and carcinoma (day 85), but not at early colitis stage (day 19) (Figure 1C and 1D).

The activation status of the macrophages in colonic tissue was also assessed by flow cytometry. The populations of pan-macrophages and M2 macrophages were determined by F4/80 and CD206 (also called mannose receptor C type 1, Mrc1) markers. Similar to the results of the immunohistochemical study, F4/80^+^ macrophages were increased after CAC challenge, and embelin significantly decreased the number of macrophages at colitis and tumor stages (Figure 1E). Moreover, F4/80^+^CD206^+^ M2 macrophages increased more than 2-fold in the colons of CAC mice after dysplasia was detectable (day 45 and 85) versus mice with colitis on day 19, and embelin effectively inhibited the augment of these M2 macrophages (Figure 1F and 1G). We next investigated mRNA expression of representative M2 genes in the colonic tissues of CAC-bearing mice. As shown in Figure 1H, mRNA of Arginase1, CD206, Trem2 (triggering receptor expressed on myeloid cells 2) and Ym1, which are typical M2 markers, were significantly reduced upon embelin treatment at carcinoma stage (day 85). Taken together, these results suggested that embelin attenuates M2-like polarization of macrophages in CAC tumors.

**Figure 2:**
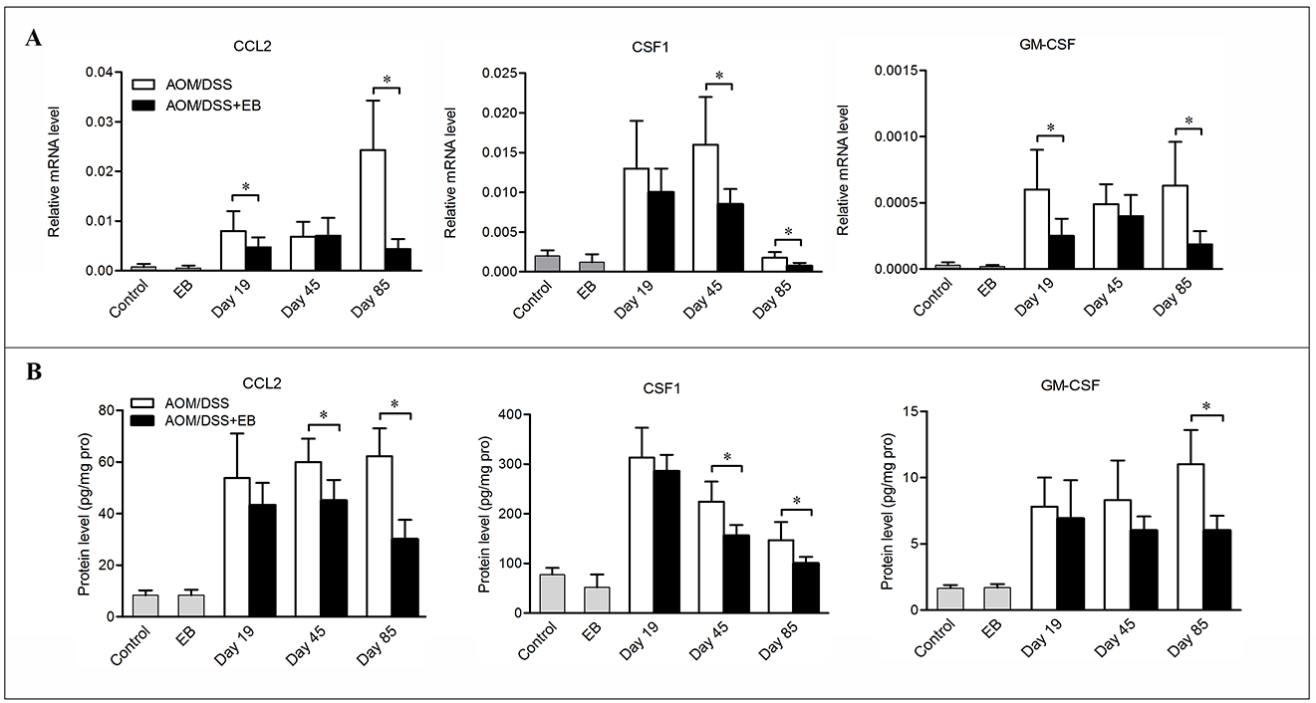
Embelin decreased chemokines expression in CAC model Colon tissues were collected at days 19, 45 and 85 after CAC challenge and mRNA (**A**) and protein (**B**) levels of CCL2, CSF-1 and GM-CSF were determined by qPCR and ELISA. Data are expressed as mean ± SD (*n* = 6–8/group; *p < 0.05).

We further examined the effects of embelin on the chemoattractants that support the recruitment and survival of macrophages. Indeed, CAC mice treated with embelin showed dramatic reduction in the mRNA and protein levels of chemokine (C-C motif) ligand 2 (CCL2), colony stimulating factor1 (CSF1) and granulocyte-macrophage CSF (GM-CSF) in colon relative to the untreated mice, and the difference was especially obvious at the carcinoma stage (Figure 2A and 2B). Such a trend was consistently associated with the decreased number of macrophages present in the tumors, suggesting that embelin blocks the recruitment of macrophages during CAC development, which may be responsible for its anti-inflammatory and anti-tumor activities.

### Embelin altered the cytokine milieu in the colon of CAC mice

**Figure 3:**
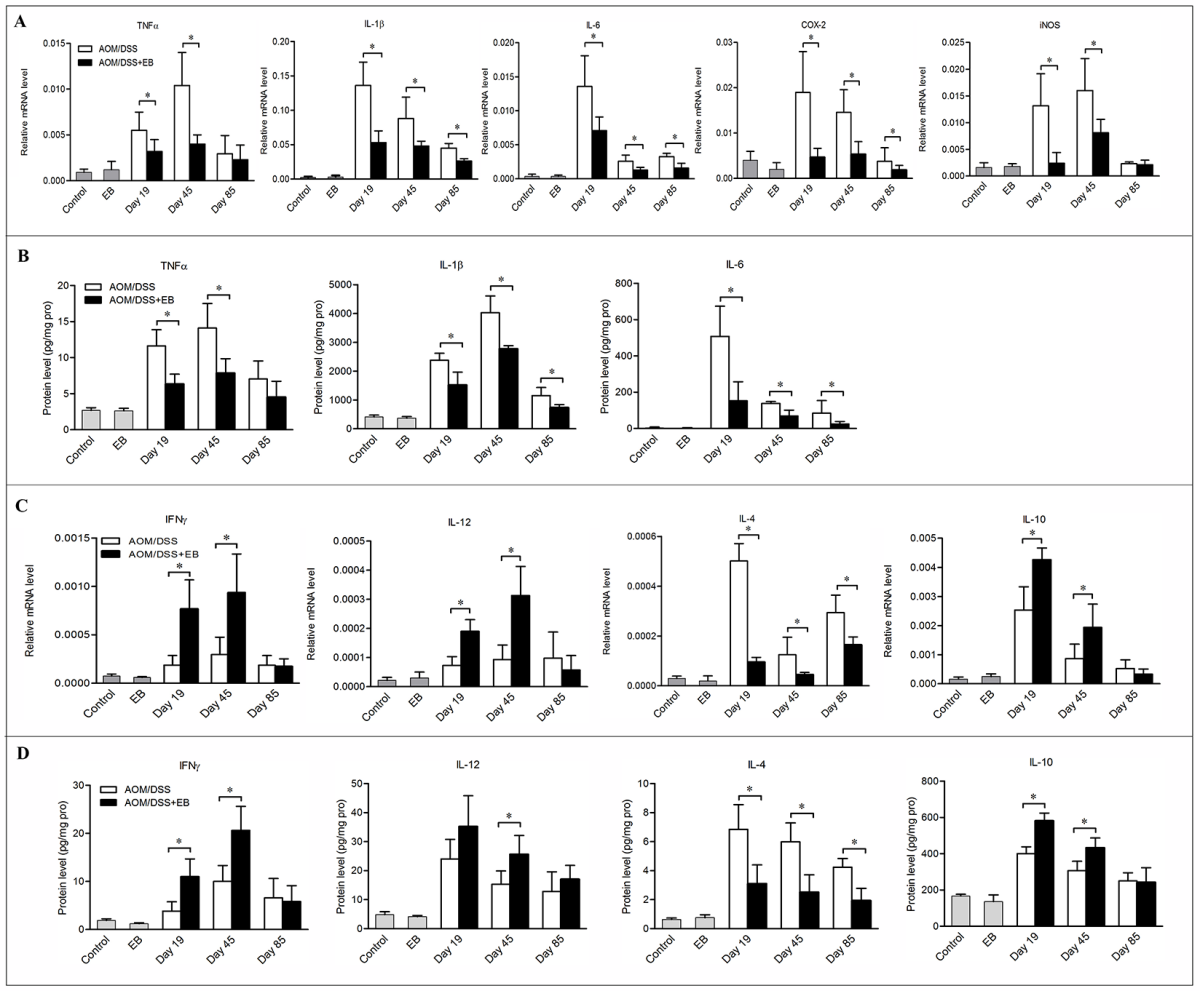
Embelin altered the cytokine milieu in the colon of CAC mice The mRNA expression (**A**, **C**) and protein level (**B**, **D**) of cytokines in the colon tissues of mice exposed to AOM/DSS for 19, 45 and 85 days were measured by qPCR and ELISA. Data are expressed as mean ± SD (*n* = 6–8/group; *p < 0.05).

We next explored the pro-inflammatory cytokines and some tumorigenic factors produced by macrophages at different stages of CAC. Consistent with the diminished infiltration of macrophages in embelin-treated mice, the mRNA levels of colonic expression of IL-1β, IL-6 and COX-2 were markedly down-regulated in these animals at days 19, 45 and 85 (Figure 3A). However, decreased mRNA expression of TNFα and iNOS was only found at days 19 and 45, but not day 85 (Figure 3A). Similarly, the protein levels of TNFα, IL-1β and IL-6 were significantly decreased by embelin administration (Figure 3B). Given the association of tumorigenesis with enhanced production of pro-inflammatory cytokines in CAC mice, our results suggested that embelin might exert its anti-tumor effect through suppressing key inflammatory signaling pathways.

As the phenotypes of macrophages may change in response to the microenvironment signals, we analyzed the cytokines that drive macrophage M1/M2 polarization in colon tissues. Treatment of CAC mice with embelin led to increased mRNA and protein expression of IFNγ and IL-12 (which are believed to be responsible for driving M1 polarization and anti-tumor activity) in the colonic mucosa, although statistic significance was only noted at days 19 and 45, but not at day 85 (Figure 3C and 3D). On the other hand, IL-4, which induces M2 phenotype, was significantly decreased by embelin at all time points. However, the impact of embelin on the expression of IL-10, another important cytokine setting macrophages in M2 polarization, followed a similar trend to that of IFNγ and IL-12 (Figure 3C and 3D). Collectively, these findings indicated that embelin alters cytokine profile in the microenvironment, which might reverse the M2 phenotype of TAMs during the CAC development.

### Embelin decreased vascularity and MMP2 expression in the late stage of colonic carcinogenesis

**Figure 4:**
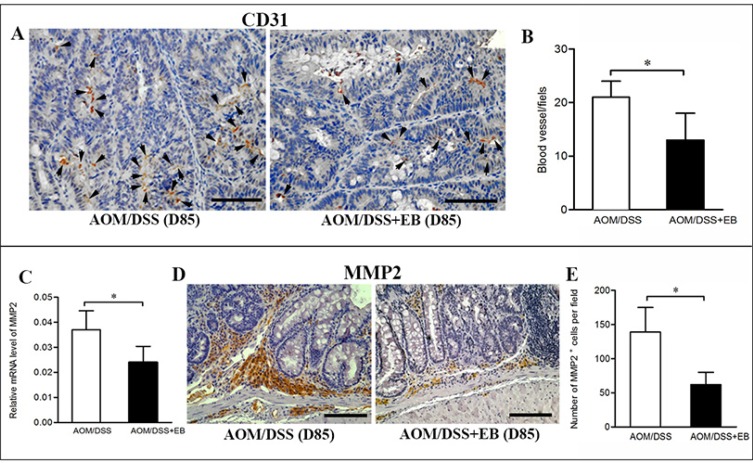
Embelin decreased vascularity and MMP2 expression in late carcinoma stage (**A**) Vascular density in colonic tumors from mice treated with or without embelin at day 85 was evaluated by immunostaining with anti-CD31 antibody. Scale bar: 50 μm. Data were quantified by blind counting of blood vessels per microscopic field (**B**). Relative mRNA expression of MMP2 in colonic tumor was analyzed by qPCR (**C**), and representative immunostaining of MMP2 in colonic tumor at day 85 after AOM/DSS exposure was shown (**D**). Scale bar: 50 μm. E. The number of MMP2^+^ cells per field was quantified (*n* > 10 fields/group). Data are expressed as mean ± SD (*n* = 6–8/group; *p < 0.05).

TAMs promote angiogenesis in tumors, therefore we assessed the impact of embelin on vascular density by CD31 immunostaining. As shown in Figure 4A and 4B, rich expression of CD31 was clearly demonstrated in the malignant colonic tissues of the CAC mice (day 85), and treatment with embelin led to a significant reduction in the vessel density and size.

Matrix metalloproteinases (MMPs) produced by TAMs, such as MMP9 and MMP2 are required for the tumor cell invasion in colon cancer [21]. As shown in Figure 4C, embelin significantly reduced the colonic expression of MMP2 mRNA in the CAC-bearing mice. Similarly, embelin treated tumor exhibited decreased expression of MMP2 in stromal cells based on immunostaining (Figure 4D and 4E). Embelin did not affect the expression of MMP9 (data not shown). Thus, embelin appears to inhibit angiogenesis and MMP2 expression, and thereby limiting tumor progression.

### Embelin significantly inhibited lipopolysaccharide-induced macrophage activation and negatively regulated NF-κB signaling in macrophages

**Figure 5:**
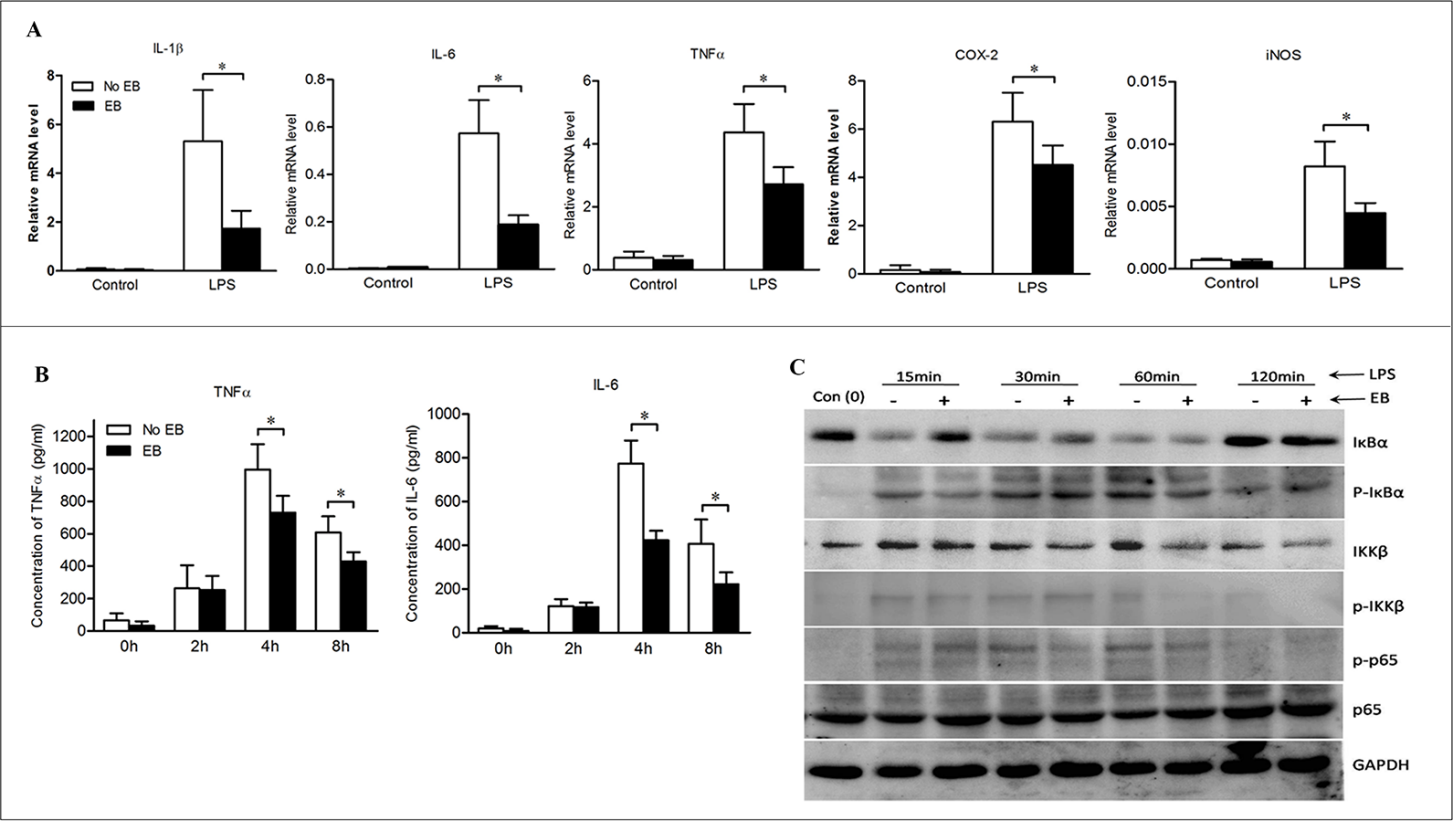
Embelin negatively regulated NF-κB signaling in macrophages (**A**) Bone marrow-derived macrophages (BMDMs) were cultured and stimulated with LPS (500 ng/ml) in the absence or presence of embelin (20 μmol/L). Macrophages were collected at 6 h and mRNA was isolated for qPCR analysis of IL-1β, TNFα, IL-6, COX-2 and iNOS. (**B**) TNFα and IL-6 in cell culture supernatant at indicated time points after LPS stimulation were measured by ELISA. (**C**) Cell lysates collected at indicated time points were analyzed for p-IκBα, IκBα, p-IKKβ, IKKβ, p-p65 and p65 by Western blot. Data are expressed as mean ± SD. *p < 0.05. All results shown are representative of three independent experiments.

To validate the direct effects of embelin on macrophage activation, bone marrow-derived macrophages (BMDMs) were cultured and stimulated with lipopolysaccharide (LPS) in the absence or presence of embelin. LPS is known to induce inflammatory responses primarily *via* the TLR4/NF-κB pathway [22]. As shown in Figure 5A, embelin strongly inhibited LPS-induced activation of BMDMs, as indicated by a significant decrease in the mRNA expression of NF-κB target genes IL-1β, IL-6, TNFα, COX-2 and iNOS. Moreover, embelin inhibited the release of TNFα and IL-6 from LPS-induced BMDMs. The inhibitory effect was most prominent after 4 h of LPS stimulation when the production of TNFα and IL-6 were suppressed by 27% and 45%, respectively (Figure 5B).

The activation of NF-κB was further analyzed by Western blot. As shown in Figure 5C, both degradation and phosphorylation of IκBα were inhibited by embelin over a range of time points (15 min to 1 h after LPS exposure). Furthermore, embelin inhibited LPS-induced phosphorylation of IKKβ and decreased IKKβ expression in BMDMs. Embelin also slightly suppressed the phosphorylation of NF-κB p65 without affecting the expression of total p65 protein (Figure 5C). These results demonstrate that embelin inhibits NF-κB signaling in macrophages, thereby reducing several key pro-inflammatory mediators involved in colitis and the associated tumorigenesis.

### Embelin reduced M2 macrophage polarization in the presence of Th2 cytokines

**Figure 6:**
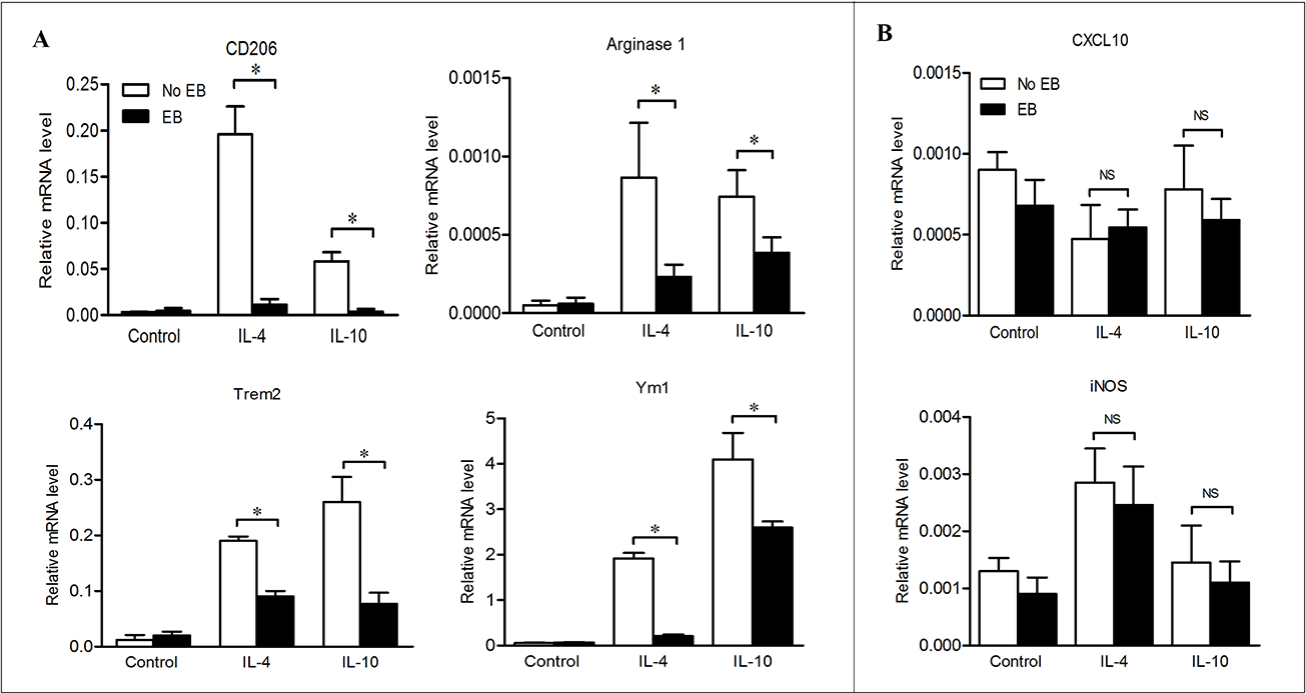
Embelin reduced M2 macrophage polarization in the presence of Th2 cytokines BMDMs were exposed to IL-4 or IL-10, in the presence or absence of embelin as described in Materials and Methods. M2 genes CD206, Arginase1, Trem2 and Ym1 (**A**) and M1 genes CXCL10 and iNOS (**B**) were analyzed by qPCR. Data are expressed as mean ± SD. *p < 0.05. NS, not significant. All results shown are representative of three independent experiments.

With the *in vivo* data, we hypothesized that embelin exerts its anti-tumor effects by impairing the survival of TAMs or changing their phenotypes. We addressed this issue by examining the impact of embelin on apoptosis and phenotype changes of macrophage *in vitro*. Treatment of BMDMs with 30 μmol/L of embelin (a concentration that could lead to significant apoptosis in colon cancer cells) failed to induce apoptosis as detected by AnnexinV/PI staining (data not shown). To explore how embelin regulated M2 macrophage polarization, BMDMs were exposed to Th2 cytokines IL-4 or IL-10 in the presence or absence of embelin, and the mRNA expression of multiple M2 and M1 genes were examined by qPCR. As shown in Figure 6A, exposure of BMDMs to IL-4 and IL-10 led to a significant increase in the expression of M2 genes including CD206, Arginase1, Trem2 and Ym1, suggesting BMDMs underwent a polarization to M2 phenotype. Importantly, 20 μmol/L of embelin dramatically reversed the patterns of M2 genes expression induced by IL-4 and IL-10 without corresponding up-regulation of the M1 genes CXCL10 and iNOS (Figure 6B). Similar results were obtained using the mouse peritoneal macrophages to analyze the expression profiles of these genes (data not shown). Collectively, these results indicated that embelin reduces M2 macrophage polarization in the presence of Th2 cytokines, which is consistent with our *in vivo* finding that TAMs lose M2 polarization after embelin treatment.

## DISCUSSION

In this study, we have demonstrated that embelin significantly depletes colon macrophages in CAC model by blocking their recruitment. Moreover, embelin may alter the expression pattern of cytokines and cause TAMs in CAC tumors to lose M2 polarization. Embelin was found to potently and negatively regulate NF-κB signaling in macrophages and inhibit the production of key pro-inflammatory mediators involved in CAC tumorigenesis, such as TNFα, IL-6 and COX-2. Furthermore, embelin could reduce M2 macrophage polarization *in vitro* even in the presence of Th2 cytokines. These results suggest that macrophages are likely the therapeutic target for embelin to exert its anti-inflammatory and anti-tumor activities during CAC development.

Macrophages in the lamina propria play a key role in the pathogenesis of IBD in human [23] and chronic colitis in rodents [24]. In CAC model, the tumorigenic functions of TAMs rely on the production of reactive oxygen species (ROS), nitrogen species (i.e., nitric oxide, NO) and a wide range of pro-inflammatory cytokines, all of which may cause mutations and promote growth of the epithelial cells [10, 11]. In addition, macrophages are known to be a major source of COX-2 production in human and mouse intestinal tumors [25, 26]. In our present time-course study, we have shown that embelin-mediated inhibition of CAC tumorigenesis relied on its inhibitory effect on the ongoing colitis. The potent anti-inflammatory and anti-tumor effects of embelin were demonstrated by the findings that this agent dramatically reduced the infiltration of macrophages and decreased the expression of TNFα, IL-6, IL-1β, COX-2 and iNOS in colonic tissues.

In solid tumors, the main source of TAMs is circulating monocytes rather than proliferating resident macrophages inside tumors. Monocytes in bone marrow are derived from myeloid progenitors, and they can enter tumors through blood circulation and subsequently differentiate into macrophages [27]. Chemotaxis, differentiation and growth of macrophages are regulated by chemoattractants including CSF1, CCL2 and GM-CSF. As demonstrated in several malignancies including CRC, overexpression of CSF1 and CCL2 may result in accelerated tumor progression and poor prognosis [28]. Moreover, CCL2 has been identified as a crucial mediator of the initiation and progression of CAC in mice [29]. A recent study reported that depletion of TAMs from the tumor tissues by an anti-CSF-1R antibody may hold promise in the treatment of some solid tumors [30]. Our data showed that treatment with embelin led to a significant impairment in the expression of CSF1, CCL2 and GM-CSF in tumor tissues whereas only very mild apoptosis in macrophages was detected. Thus, we speculate that the macrophage deletion by embelin in the colon tissues of CAC model is attributed to blocking of recruitment rather than induction of apoptosis.

TAMs usually exhibit an M2-like phenotype, which is associated with pro-tumorigenic functions [12, 14]. Our data demonstrated that embelin resulted in a decreased infiltration of M2 macrophages in the tumor tissue. This finding was further demonstrated in the *in vitro* study where Th2 cytokines-induced M2 polarization can be directly reversed by embelin. TAMs are strongly influenced by the tumor microenvironment. IFNγ is a canonical M1-polarizing cytokine and it prevents tumor development and reverses the M2 phenotype of TAMs [13]. Up-regulation of IFNγ is associated with improved survival of CRC patients [31]. IFNγ is also a key molecule in IL-12-mediated anti-tumor activity, as IL-12 can stimulate the secretion of IFNγ by natural killer cells and T cells [32]. On the other hand, IL-4 plays a regulatory role on macrophage polarization towards the tumor-promoting phenotype [33]. We observed that embelin led to an up-regulation of IFNγ and IL-12, and a down-regulation of IL-4, suggesting that in addition to its direct effect, this agent changed the phenotypes of TAMs in CAC tumors through altering the microenvironment. Unexpectedly, we observed an up-regulation of IL-10, another important M2-polarizing cytokine, in embelin-treated mice. IL-10 is known to be a potent anti-inflammatory cytokine in colitis and may exert a tumor suppressive role in CRC [34]. Increased IL-10 expression was noted in the colonic tissues of mice at the stages of acute and chronic colitis, which we believe could be partly responsible for the anti-inflammatory activity of embelin. As many cytokines within the CAC microenvironment are involved in macrophage activation, these factors need to be assessed together to understand macrophage polarization and the underlying mechanisms *in vivo*.

The canonical IKKβ/NF-κB pathway provides the critical mechanistic link between inflammation and cancer [35]. Microbial and viral infections and pro-inflammatory cytokines trigger this pathway through activating the IκB kinase (IKK) complex. The involvement of the IKKβ-dependent NF-κB activation is well-established in inflammation and cancer in CAC model [10]. We confirmed that embelin potently suppressed LPS-induced activation of canonical NF-κB signaling in macrophages and correspondingly reduced the expression of NF-κB target genes, including IL-1β, IL-6, TNFα, COX-2 and iNOS. We previously reported that embelin inhibits NF-κB activation and induces apoptosis in colon cancer cells [19]. Here, our results indicated that embelin may exert its anti-tumor effect in CAC through the different mechanism in epithelia cells and macrophages. While in epithelia cells embelin limited tumor growth by increasing apoptosis, in macrophages it attenuated production of inflammatory mediators that promote tumor initiation and promotion. LPS-induced activation of NF-κB is mediated by TLR4 [36], which is known to be critical in mediating the activation of macrophages in the CAC model [37]. Our data also showed that embelin exerted a similar effect on macrophages as the TLR4 inhibitor TAK-242 [38], suggesting that the inhibitory effect of embelin on NF-κB pathway may be mediated by dampening TLR4.

Strategies leading to macrophage depletion have been demonstrated to reduce angiogenesis and tumor growth in the xenograft tumor models [39]. Here, we found that embelin decreased angiogenesis and MMP2 expression within tumor tissues at late carcinoma stage, suggesting that attenuation of M2 polarization of TAMs may partially contribute to the inhibition of tumor progression by embelin as our previously reported [20]. Based on the TAMs-depleting and re-education effects of embelin, we may speculate that combination of embelin with conventional treatments such as chemotherapy or immunotherapy might exert synergistic effects in cancer therapy.

In conclusion, we have demonstrated that macrophage targeting is a key component of the anti-inflammatory and anti-tumor properties of embelin. In addition to blocking macrophage recruitment, embelin changes TAM phenotype within the tumor microenvironment and eliminates their tumor-promoting functions during pathogenesis of CAC. The current results strengthen the rationale for future validation of embelin in the prevention and treatment of CAC.

## MATERIALS AND METHODS

### Induction of colitis-associated colon cancer

The CAC model was generated as we described previously [20] using azoxymethane (AOM; Sigma-Aldrich, St. Louis, USA) and dextran sulfate sodium (DSS; Affymetrix, Santa Clara, USA). Briefly, male C57BL/6 mice (6–8 weeks) were injected with two doses of AOM (10 mg/kg and 5 mg/kg, respectively), followed by three cycles of 2% DSS administration (in the drinking water). Embelin (50 mg/d/kg body weight; Advance Scientific & Chemical, Inc.) or vehicle (dimethylsulfoxide) was added to the diet and given to mice 10 days before the CAC challenge, and then continued until harvest. The animals were sacrificed at the indicated time intervals for macroscopic inspection, histological analysis, and total RNA extraction. All animal experiments were approved by the Animal Studies Committee of Peking University First Hospital, China.

### Histopathological and immunohistochemical analyses

Paraffin-embedded sections were cut at 4 μm thickness, stained with hematoxylin and eosin (H & E), and examined by a pathologist blinded to the experimental groups. The extent of colitis was scored according to the protocols described previously [40]. Immunohistochemical analysis was performed as we described [20], using the following primary antibodies: anti-CD68, anti-MR (all from Abcam, Cambridge, UK), anti-CD31 and anti-MMP2 (Santa Cruz, Dallas, USA).

### Isolation of immunocytes from colonic tissues and flow cytometry analysis

Colonic tissues were cut into small pieces (1–2 mm) and incubated in RPMI 1640 medium containing 1 mg/ml collagenase IV, 0.1 mg/ml hyaluronidase and 600 U/ml DNase I (all from Sigma-Aldrich, USA) for 1 h at 37°C with frequent agitation. After digestion, the cell mixture was filtered through a 70 μm strainer, collected and washed in PBS. Subsequently, the pellets were resuspended in RPMI 1640 and then layered on a percoll density gradient to isolate immunocytes. The freshly isolated cells were stained with antibodies for 30 minutes at 4°C, washed and then analyzed on a BD Influx^™^ (BD Biosciences, USA). The following monoclonal anti-mouse antibodies were used: anti-Gr-1-PE-Cy7, anti-F4/80-PE (eBioscience, CA, USA) and anti-CD206-Alexa Fluro 647 (BD Pharmingen, USA). In parallel, cells were stained with the respective control isotype antibodies. The acquired data were analyzed using Flowjo software.

### Generation of macrophages and *in vitro* assays

Mouse macrophages were derived as described elsewhere [14]. Briefly, bone marrows were collected from the femur and tibia of C57BL/6 mice. The marrow cells were cultured in DMEM medium supplemented with 10% FBS, 1% penicillin-streptomycin (all from Invitrogen), and 20 ng/ml recombinant mouse M-CSF (PeproTech, Rocky Hill, USA) for 5 days to differentiate into macrophages. For analysis of macrophage polarization, cells were cultured with 20 ng/ml of mouse recombinant IL-4 or 20 ng/ml of mouse recombinant IL-10 (PeproTech) for 24 h, together with or without 20 μmol/L of embelin. For macrophage activation and NF-κB signaling assays, cells were pretreated with embelin for 1 h, and then stimulated with LPS (500 ng/ml, Sigma-Aldrich) for indicated durations.

### Measurement of cytokine levels by ELISA

The levels of cytokines in the supernatant and colonic tissues were measured by ELISA using commercial kits (R & D Systems and eBioscience) according to the manufacturer's instructions. The levels of IL-6 and TNFα in supernatant were expressed in pg/mL. Cytokines in the colonic tissue homogenate were expressed as picogram per milligram of protein (pg/mg pro).

### Western blot and quantitative real time PCR (qPCR) assays

Immunoblotting was performed as we previously described [19, 20], using the following primary antibodies: anti-glyceraldehyde-3-phosphatedehydrogenase (GAPDH, Abcam), anti-phospho-IκBα (Ser32), anti-IκBα, anti-phospho-IKKβ (Ser181), anti-IKKβ, anti-phospho-NF-κB p65 (Ser536) and anti-NF-κB p65 (all from Cell Signaling Technology, Danvers, USA). The sequences of primers used in qPCR are listed in Supplementary Data, Table S1.

### Statistical analysis

Data are expressed as mean ± SD. Statistical significance was determined by Student's *t* test, and a *p* < 0.05 was considered statistically significant. Statistics were performed using SPSS 10.0 software.

## SUPPLEMENTARY MATERIALS FIGURES AND TABLES


