# Supplementary material for: Macrophage targeting contributes to the inhibitory effects of embelin on colitis-associated cancer
Source: Oncotarget. 2016 Jan 21;7(15):19548–58. doi: 10.18632/oncotarget.6969 (PMC4991400; doi:10.18632/oncotarget.6969)
Supplement: Supplementary file 1 [file oncotarget-07-19548-s001.pdf]

Macrophage targeting contributes to the inhibitory effects of embelin on colitis-associated cancer

Supplementary Materials

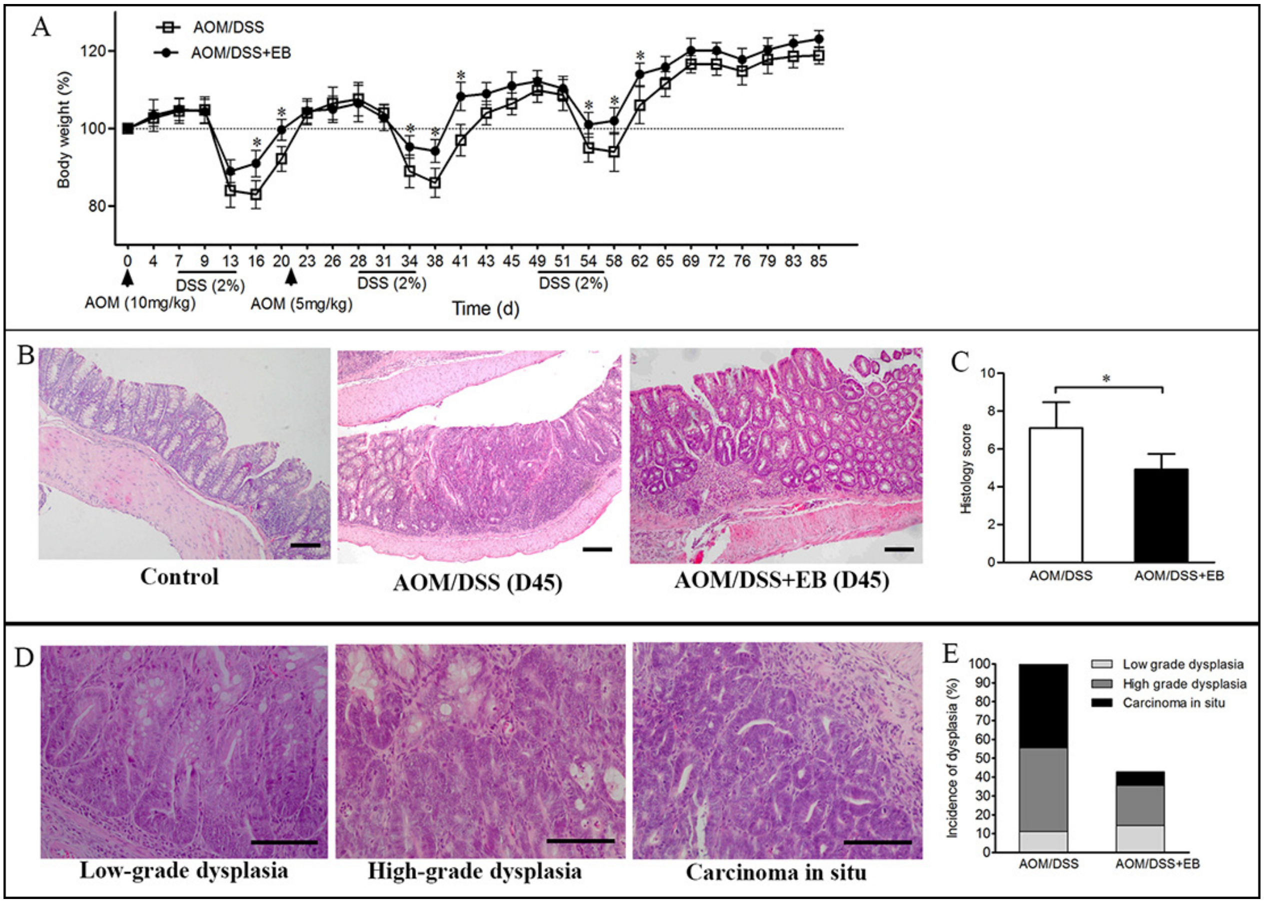

Supplementary Figure S1: Embelin (EB) protected mice against colitis and colitis-associated dysplasia during the early stage of CAC. (**A**) Body weight changes throughout the study. (**B**) Representative images of H & E staining of colon section at day 45 after AOM/DSS administration. Scale bar: 50 µm. (**C**) Semiquantitative scoring of histopathology. (**D**) Representative high magnification images showing dysplasia in colon tissue at day 45 of CAC regimen. Scale bar: 50 µm. (**E**) Overall grading of dysplasia in each group. Data are expressed as mean ± SD (n = 10–14/group; $*p < 0.05$).

Supplementary Table S1: The sequences of primers used in qPCR assay

Genes	Sequences	
Arginase1	Forward	5′-CTCCAAGCCAAAGTCCTTAGAG-3′
	Reverse	5′-AGGAGCTGTCATTAGGGACATC-3′
Ccl2	Forward	5′-TTAAAAACCTGGATCGGAACCAA-3′
	Reverse	5′-GCATTAGCTTCAGATTTACGGGT-3′
Cox2	Forward	5′-TGAGCAACTATTCCAAACCAGC-3′
	Reverse	5′-GCACGTAGTCTTCGATCACTATC-3′
Csf1	Forward	5′-ATGAGCAGGAGTATTGCCAAGG-3′
	Reverse	5′-TCCATTCCCAATCATGTGGCTA-3′
Cxcl10	Forward	5′-CCAAGTGCTGCCGTCATTTTC-3′
	Reverse	5′-GGCTCGCAGGGATGATTTCAA-3′
Gm-csf	Forward	5′-GGCCTTGGAAGCATGTAGAGG-3′
	Reverse	5′-GGAGAACTCGTTAGAGACGACTT-3′
Ifnγ	Forward	5′-ATGAACGCTACACACTGCATC-3′
	Reverse	5′-CCATCCTTTTGCCAGTTCCTC-3′
Il-1β	Forward	5′-GCAACTGTTCCTGAACTCAACT-3′
	Reverse	5′-ATCTTTTGGGGTCCGTCAACT-3′
Il-4	Forward	5′-GGTCTCAACCCCCAGCTAGT-3′
	Reverse	5′-GCCGATGATCTCTCTCAAGTGAT-3′
Il-6	Forward	5′-TAGTCCTTCCTACCCCAATTTCC-3′
	Reverse	5′-TTGGTCCTTAGCCACTCCTTC-3′
Il-10	Forward	5′-GCTCTTACTGACTGGCATGAG-3′
	Reverse	5′-CGCAGCTCTAGGAGCATGTG-3′
Il-12	Forward	5′-ACTCTGCGCCAGAAACCTC-3′
	Reverse	5′-CACCCTGTTGATGGTCACGAC-3′
iNos	Forward	5′-GTTCTCAGCCCAACAATACAAGA-3′
	Reverse	5′-GTGGACGGGTCGATGTCAC-3′
Mmp2	Forward	5′-ACCTGAACACTTTCTATGGCTG-3′
	Reverse	5′-CTTCCGCATGGTCTCGATG-3′
Mrc1	Forward	5′-CTCTGTTCAGCTATTGGACGC-3′
	Reverse	5′-CGGAATTTCTGGGATTCAGCTTC-3′
Tnfα	Forward	5′-CCCTCACACTCAGATCATCTTCT-3′
	Reverse	5′-GCTACGACGTGGGCTACAG-3’
Trem2	Forward	5′-CTGGAACCGTCACCATCACTC-3′
	Reverse	5′-CGAAACTCGATGACTCCTCGG-3′
Ym1	Forward	5′-CAGGTCTGGCAATTCTTCTGAA-3′
	Reverse	5′-GTCTTGCTCATGTGTGTAAGTGA-3′
Gapdh	Forward	5′-AGGTCGGTGTGAACGGATTTG-3′
	Reverse	5′-TGTAGACCATGTAGTTGAGGTCA-3′
